# Comparing the permeability of human and porcine small intestinal mucus for particle transport studies

**DOI:** 10.1038/s41598-020-77129-4

**Published:** 2020-11-20

**Authors:** Lukasz Krupa, Balazs Bajka, Robert Staroń, Didier Dupont, Harjinder Singh, Krzysztof Gutkowski, Adam Macierzanka

**Affiliations:** 1 Department of Gastroenterology and Hepatology with Internal Disease Unit, Teaching Hospital No. 1, Chopina 2, 35-055 Rzeszów, Poland; 2 Department of Nutritional Sciences, King’s College London, London, SE1 9NH UK; 3 STLO, INRAE, Agrocampus Ouest, 35000 Rennes, France; 4 Riddet Institute, Massey University, Private Bag 11 222, Palmerston North, 4442 New Zealand; 5 Department of Colloid and Lipid Science, Faculty of Chemistry, Gdańsk University of Technology, Narutowicza 11/12, 80-233 Gdańsk, Poland

**Keywords:** Permeation and transport, Gastrointestinal models, Small intestine, Biophysical chemistry, Drug delivery

## Abstract

The gastrointestinal mucus layer represents the last barrier between ingested food or orally administered pharmaceuticals and the mucosal epithelium. This complex gel structure plays an important role in the process of small intestinal absorption. It provides protection against hazardous particles such as bacteria but allows the passage of nutrients and drug molecules towards the intestinal epithelium. In scientific research, mucus from animal sources is usually used to simulate difficult-to-obtain human small intestinal mucus for investigating the intramucus transport of drug delivery systems or food nanoparticles. However, there is a lack of evidence the human mucus can be reliably substituted by animal counterparts for human-relevant transport models. In this report, a procedure for collecting human mucus has been described. More importantly, the permeability characteristics of human and porcine small intestinal mucus secretions to sub-micron sized particles have been compared under simulated intestinal conditions. Negatively charged, 500 nm latex beads were used in multiple-particle tracking experiments to examine the heterogeneity and penetrability of mucus from different sources. Diffusion of the probe particles in adult human ileal mucus and adult pig jejunal and ileal mucus revealed no significant differences in microstructural organisation or microviscosity between the three mucus types (*P* > 0.05). In contrast to this interspecies similarity, the intraspecies comparison of particle diffusivity in the mucus obtained from adult pigs vs. 2-week old piglets showed better penetrability of the piglet mucus. The mean Stokes–Einstein viscosity of the piglet jejunal mucus was approx. two times lower than the viscosity of the pig jejunal mucus (*P* < 0.05). All mucus structures were also visualised by scanning electron microscopy. This work validates the use of porcine small intestinal mucus collected from fully-grown pigs for studying colloidal transport of sub-micron sized particles in mucus under conditions mimicking the adult human small intestinal environment.

## Introduction

In recent years, scientific literature has given more and more attention to defining the physiological functions, biochemical composition and microstructural organisation of the small intestinal mucus layer^1–5^. This is largely because the mucus secretion can be considered either as advantageous or disadvantageous in terms of regulating transport of molecules and particles towards the intestinal epithelium^6,7^. One of the major macromolecular components of the small intestinal mucus are mucin glycoproteins (mainly MUC2) secreted by epithelial goblet cells^8^. Together with extracellular DNA originating from epithelial cell turnover, mucins are responsible for the viscoelastic character of the mucus gel^9^. By covering the epithelial surface, the mucus gel protects mucosal tissue from abrasion that can be caused by the peristaltic movement of food digested in the gut, and from direct exposition to luminal pathogens. Conversely, the protective mucus layer is a barrier that nutrients released from digested food or the pharmaceuticals delivered to the intestinal lumen need to cross in order to get absorbed by the underlying epithelial enterocytes. These contrasting properties are crucial in the small intestine where absorption of the majority of nutrients and orally administered pharmaceuticals takes place, and where the mucus layer is thinnest^10–12^.

The physiology of the human gastrointestinal (GI) tract is historically considered most similar to that of pigs^13,14^. This has led to porcine digestive enzymes, mucins and bile being widely used in in vitro digestion models aiming to simulate the physiology of the human gut. However, the composition of bovine bile has been found to be closest to that in humans^15^. Consequently, the recently published methods for standardised in vitro static and semi-dynamic simulations of gastrointestinal food digestion^16,17^ have recommended the use of bovine bile over porcine bile for mimicking the human physiological profile of bile salts during the small intestinal digestion more closely. This example shows that any analogies in the pig and human GI physiology should not be accepted without scientific validation. Nevertheless, porcine small intestinal mucus is frequently used in studies aiming to simulate interactions of particles and molecules (e.g. drug nanocarriers, food digesta, bioactive/pharmaceutical molecules, etc.) with mucus in the human small intestine^18–21^, despite the permeability and barrier properties of human and porcine mucus not having been scientifically compared before. This may undermine the human relevance of any results obtained with porcine mucus.

A recent study^22^ has shown that removing mucus from the mucosal tissue of porcine jejunum does not considerably affect the microstructure of mucus with regard to the diffusivity of penetrating particles. The microviscosity of the collected mucus was very similar to the microviscosity of the intact mucus attached to mucosal tissue. Importantly, the storage of the collected mucus, which involved freezing and thawing, did not change its microviscosity and permeability to particles. These findings might be very useful in terms of convenient planning and performing of mucus penetration studies that aim to reflect physiological transport of nutrients or orally administered pharmaceuticals towards the mucosal epithelium in the human small intestine. However, the study did not actually investigate whether the transport characteristics in porcine mucus are similar to those that can be expected in the human small intestinal mucus. This presents a substantial gap in the physiological relevance of any studies solely using porcine mucus to mimic human conditions. The aim of this work was to compare the permeability of human and porcine small intestinal ex vivo mucus samples (i.e. the mucus collected from the surface of the mucosal tissue) to sub-micron sized particles in order to provide a recommendation on whether the porcine mucus can be used as a valid substitute of the human mucus in in vitro models mimicking the intramucus colloidal transport under human small intestinal conditions.

## Experimental

### Human small intestinal mucus collection

The studies on the human small intestinal mucus were approved by the ethics committee of the Regional Medical Chamber in Rzeszów, Poland (certificate no. 4/B/2015). All methods were planned and conducted in accordance with the ethical principles outlined in the Declaration of Helsinki. Aspiration of mucus from a terminal ileum was done during the diagnostic colonoscopy performed at the Teaching Hospital No 1 in Rzeszów. The procedure duration was extended for a maximum of 5 min required to obtaining mucus samples. A typical indication for colonoscopy was either occasional lower-GI bleeding, persistent unexplained abdominal pain, or screening and surveillance of colorectal polyps, and 51 individuals (31 men and 20 women), aged 34–67 years, were initially included in the study. Any evidence of inflammatory changes to the mucosa of the colon and/or the terminal ileum confirmed during the procedure disqualified a subject from the study. All individuals who agreed to take part in the study were clearly explained about the procedure and instructed by a clinician regarding the bowel preparation. They have all provided informed consent prior to the examination. Personal information of the volunteers was de-identified.

An empty and adequately clean colon is a prerequisite of diagnostic or therapeutic colonoscopy^23^. All participants in our study followed a low residue diet protocol for 5 days prior to colonoscopy in order to improve bowel preparation. Individuals listed for colonoscopy received bowel preparation with 4 L of Fortrans (Ipsen Pharma) aqueous solution (1 sachet dissolved in 1 L of water). One sachet of Fortrans powder contained Macrogol 4000 (64 g), anhydrous sodium sulphate (5.7 g), sodium bicarbonate (1.68 g), sodium chloride (1.46 g), and potassium chloride (0.75 g). The timing of bowel preparation prior to colonoscopy has a very significant impact on the preparation quality. It has been reported that the optimal time for the colonoscopy procedure after completion of bowel preparation is 3–4 h, and should be less than 8 h after completion of the preparation^24^. This concept has led to a split-dose preparation, which requires taking a portion of the bowel preparation solution the night prior to colonoscopy (50% of the total dose) and the remaining portion on the day of colonoscopy. This method was found to significantly improve the quality of bowel preparation and patients’ compliance^25^. The split-dose preparation was used in this study. The participants received two sachets of Fortrans diluted in 2 L of water between 6:00 and 8:00 p.m. on the day before colonoscopy and the remaining 2 L of solution between 5:00 and 6:00 a.m. on the day of the procedure.

**Figure 1:**
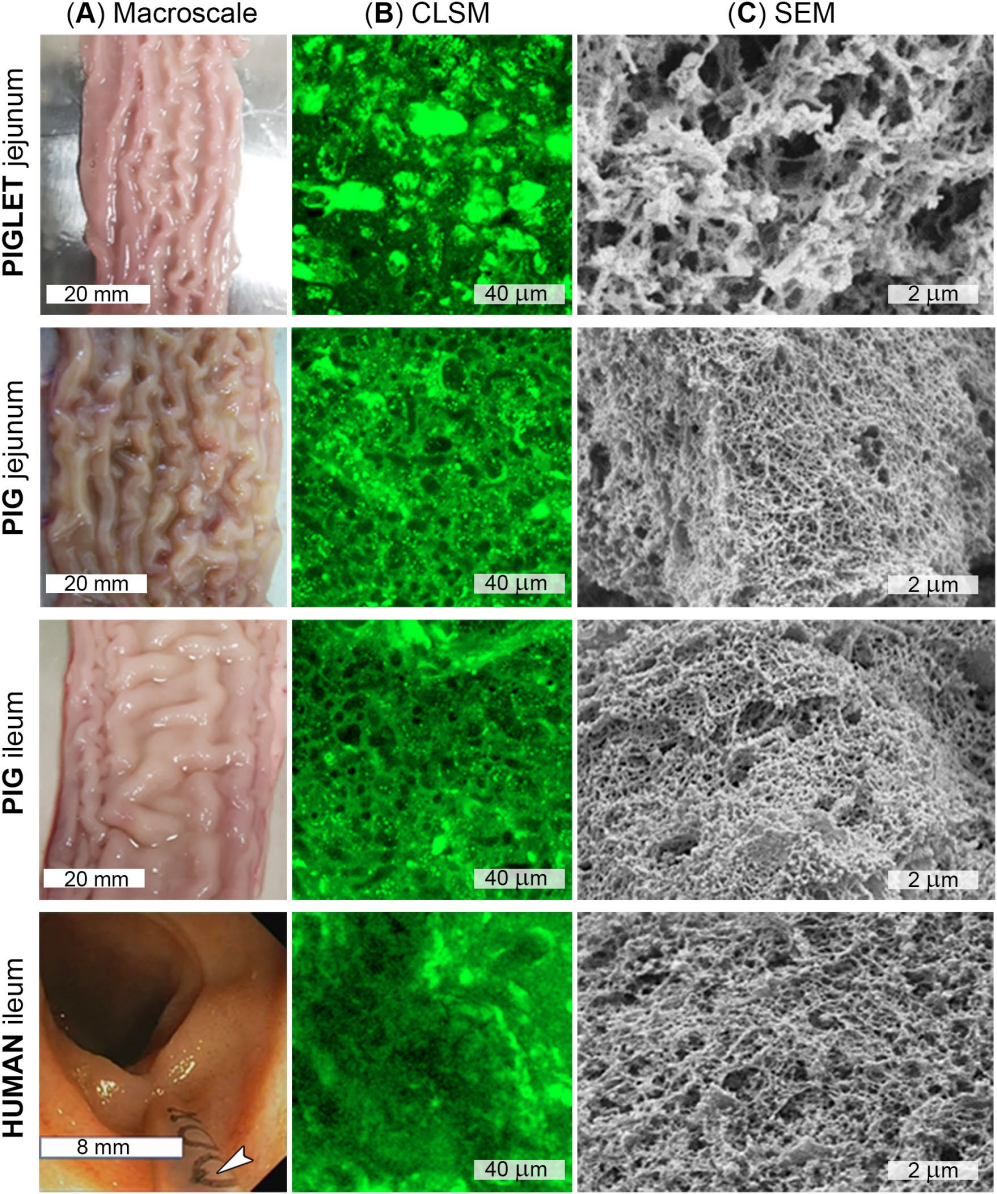
(**A**) Macroscale images of the piglet jejunal, the pig jejunal and ileal, and the human ileal mucosal surfaces. The freshly excised porcine small intestinal segments were opened along the mesenteric border in order to expose the mucosal epithelium covered with mucus. The mucosal surface of the human ileum was imaged during the colonoscopy procedure that was extended into the distal ileum. The arrow (**A**, bottom image) indicates the position of a plastic catheter used for collecting mucus. A representative video clip showing the collection of the human ileal mucus has been provided as Supplementary Video S1. (**B**) Representative confocal laser scanning microscopy (CLSM) images of mucus collected from the different mucosal surfaces (the images were created using Image-Pro Analyzer 7.0 software (Media Cybernetics, Inc.; https://www.mediacy.com/imagepro). The mucus specimens were stained for mucin with WGA-Oregon Green. (**C**) Scanning electron microscopy (SEM) of the collected mucus showing mucin polymer networks.

As a routine part of colonoscopy examination, the intubation of the terminal ileum is performed^26^. This part of the small bowel contains a relatively thin layer of the mucus which is distributed on the mucosal surface of the bowel wall. We only performed aspiration of the mucus samples from the terminal ileum in participants who had bowel preparation rated as excellent (i.e. rate 9 of the Boston Scale^27^). Once the terminal ileum was intubated with the colonoscope, the mucosal layer was inspected and an optimal area for aspiration of mucus chosen (i.e. an area free of residual liquid, etc.). At the same time, an assistant endoscopist inserted a disposable ERCP plastic catheter through the biopsy channel of the colonoscope. While inserting the catheter, air was being constantly insufflated with a syringe attached to one end of the catheter in order to prevent incidental aspiration of any material that might have resided in the biopsy channel and would potentially lead to the contamination of a sample. Once the catheter was in the lumen of the terminal ileum (Fig. 1A), the principal endoscopist was in charge of the control of the colonoscope tip and the catheter. The assistant endoscopist performed aspiration of the mucus by applying a gentle suction with a syringe. This part of the procedure has been shown in the Supplementary Material (Video S1). Mucus was only aspirated into the tip of the catheter in order to limit disturbing of the sampled mucus, and from the mucosal surface of the terminal ileum located between 5 and 25 cm from the ileocecal valve. Typically, no more than ca. 0.5 mL mucus was aspirated from one subject over the limited time that was allowed for mucus collection. Immediately after aspiration, the plastic catheter was removed and the sample gently transferred into plastic test tubes. The tubes were sealed and instantly immersed in liquid nitrogen for snap freezing. Samples were stored at − 80 °C prior to further examination.

Samples of the ileal mucus were collected from 17 individuals. This reduction from the initial number of 51 recruited individuals was due to the fact that even the slightest sign of mucosal inflammation detected during colonoscopy, and/or bowel preparation being rated below the ‘excellent’ mark, disqualified a subject. Finally, mucus samples collected from five individuals (three men and two women, 43–49 years old) were used in the experiments described below in order to narrow down the age range of adult humans.

### Porcine small intestinal mucus collection

A detailed procedure of collecting porcine mucus, including the ex vivo handling of intestinal tissue, was described previously^9,22^. In brief, mucus was gently removed with a soft-rubber scraper from the mucosal surface (Fig. 1A) of freshly excised and cleaned small intestines of (1) 7–10-month old pigs (this being referred to as the ‘adult pig mucus’ or the ‘mucus from fully-grown pigs’ throughout the paper) and (2) 2-week old piglets (this being referred to as the ‘piglet mucus’). Mucus was collected from the most proximal, 1-m-long jejunal segment (from both, adult pigs and piglets) and/or the most distal, 1-m-long ileal segment (from adult pigs only). The last 5 cm of the ileum before the ileocecal valve was discarded. Typically, 10–15 mL of the jejunal and ileal mucus was collected from one pig, or 5–8 mL of the jejunal mucus from one piglet. Aliquots of collected mucus were immediately transferred to 0.5 mL plastic screw-cap tubes for snap freezing in liquid nitrogen, and then stored at − 80 °C. The collection was carried out within 20 min from animal slaughter. Mucus was incubated for 10 min at RT before use. As reported previously^22,28^, the freezing, storing and thawing cause no substantial differences in the macro- and microrheological properties of small intestinal mucus.

As with the previously study^9^, the fresh piglet small intestines were obtained from INRA-UMR PEGASE (Physiology, Environment and Genetics for the Animal and Livestock Systems) in Saint-Gilles, France, in strict accordance with the recommendations of the French Ministry of Agriculture (Directive 2001-464 29/05/01) and EEC (Directive 86/609/EEC) for the care and use of animals in research, and under approved authorisation certificate for experiments on animals (certificate no. 7676). INRA-UMR PEGASE is a holder of the agreement for experimentation on pigs (no. A35622) authorised by the Veterinary Services of the French Ministry of Agriculture. Five piglets were reared and slaughtered in compliance with French national regulations and according to procedures approved by the French Veterinary Services at INRA-UMR PEGASE. Piglets were slaughtered by electrical stunning and exsanguination at the experimental slaughterhouse that is authorised for commercial meat production. As the piglets used in this study were not specifically included in any experimental protocol on live animals before slaughtering, there was no ethical requirement for collecting intestinal tissue samples from slaughtered animals.

The fresh adult pig small intestines^22^ were obtained from a local abattoir (H. G. Blakes Ltd, Costessey, Norfolk, UK), from five healthy animals (7–10-month old) that were slaughtered for a commercial meat production process, and therefore any ethical requirements that would be specific for this study were not necessary. The authors obtained permission from the abattoir to collect and use these samples for scientific research. Pigs were routinely fasted prior to despatch for slaughter. This largely reduced the amount of digesta residing in the intestines obtained for this study.

### Dry weight determination

The dry weight of mucus was determined according to the method described previously^9^. Samples were analysed in triplicate, and means of the results used for further analysis.

### Scanning electron microscopy

Structural imaging of the collected human and porcine mucus was conducted by scanning electron microscopy (SEM) according to the method reported previously^3^. Namely, 100 µL of mucus was pipetted into the centre of a 15 × 15 × 5 mm cryomold (Sakura Finetek Inc., Tolerance, CA, USA) containing 2% agarose (Lonza., Rockland, MA, USA) at 37–40 °C. Once set, the excess agarose was trimmed, leaving ca. 2 mm surrounding the mucus droplet. The samples were fixed with 2.5% glutaraldehyde (Agar Scientific, Stansted, UK) in 0.1 M PIPES buffer (pH 7.2) overnight. This was followed by 3 × 15 min washes with 0.1 M PIPES buffer and dehydration through a series of ethanol solutions (10, 20, 30, 40, 50, 60, 70, 80, 90, 100% × 3) for at least 15 min in each. The mucus samples were critical point dried in a Leica EM CPD300 critical point drier (Leica Microsystems, Mannheim, Germany) using liquid carbon dioxide as the transition fluid. The mucus droplets were split with a razor blade and allowed to tear apart to reveal a free-break surface. The pieces were mounted on aluminium SEM stubs using silver paint with the torn surfaces facing upwards. The samples were coated with gold in an Agar high resolution sputter-coater apparatus. SEM was carried out using a Zeiss Supra 55 VP FEG SEM (Carl Zeiss, Germany), operating at 3 kV.

### Multiple-particle tracking in mucus

Specimen preparation for confocal laser scanning microscopy (CLSM) and the particle tracking were done according to the method described recently^22^. Briefly, 500 nm red-fluorescent, carboxylate-modified latex beads (L3280, Sigma-Aldrich, Poole, UK) were used to probe permeability of mucus. The beads were incubated with bile salts (BS) before the experiment. Two BS, sodium taurocholate and sodium glycodeoxycholate (T4009 and G9910, Sigma-Aldrich, Poole, UK), were dissolved together (1:1 mol/mol) in a simulated intestinal fluid (SIF, pH 7.4) to give a total 150 mM BS stock solution. The SIF was made of an oxygenated PBS buffer (Sigma-Aldrich, Poole, UK) completed with 1 mM calcium chloride, 25 mM sodium bicarbonate, 0.02% w/v sodium azide and protease inhibitors (Roche Diagnostics GmbH, Mannheim, Germany; 1 tablet in 50 mL buffer) at pH 7.4. The BS compounds were selected to mimic the properties of BS in human bile^29,30^. The BS stock was diluted with SIF, and the latex beads added to the final concentration of 2.5 × 10^–3^% w/w. The final, total concentration of BS was 11 mM, which corresponded to an average, physiological concentration of BS in the postprandial small intestinal lumen of adult human^31^. The ζ-potential of the dispersion was obtained from dynamic light scattering measurements, using the method described before^9,22^.

Mucus samples were prepared separately. Mucus was stained at RT (5 min) for mucins with WGA-Oregon Green (W6748, Invitrogen) at the final dye concentration of 10 µg/mL, and placed in a 1-mm deep optical cell. The cell was filled to ca. 80% of its volume (ca. 40 µL of mucus was required for one specimen) and carefully covered with a coverslip, so that the coverslip was in contact with the mucus. The remaining volume was gently filled with the dilute aqueous dispersion of the latex beads through an opening in the cell, and the cell finally sealed. The procedure prevented dilution of the mucus gel with the dispersion, and allowed for simulating the transport of particles from the lumen into the mucus layer in the small intestine. Specimens were incubated for 20 min at 37 ± 0.1 °C on a temperature-controlled microscope stage. A representative image showing the beads that entered mucus matrix has been shown in the Supplementary Material (Fig. S1).

Motion of the beads in mucus was recorded at 37 ± 0.1 °C with a Leica TCS SP confocal laser scanning head mounted on a Leica DMRE upright microscope (Leica Microsystems (UK) Ltd, UK), using a 40×/1.25 NA oil-immersion objective. Specimens were scanned 30–50 μm below the coverslip, and at a temporal resolution of 1 s for 50 s. Trajectories of 110–150 beads per experiment were analysed, with no more than 30 beads analysed simultaneously in the field of view. Only those beads that managed to enter the mucus matrix during the incubation step were tracked. A representative video clip of the confocal time-lapse microscopy has been included as Supplementary Video S2 online. The video (67 × 50 µm frame) shows transport of 500 nm latex beads inside the pig jejunal mucus (i.e. after they have entered the mucus matrix) captured over the course of 150 s and displayed at 15× speed. The position of the mucus matrix was monitored during the experiments for each time series captured. This was conducted by a software-aided analysis of the mucus matrix pattern. If the post-experiment analysis detected that the matrix had drifted during the experiment, recorded time series were excluded from providing any particle-tracking data for the beads positioned inside such a drifting specimen. In specimens that did not show signs of mucus drifting, particle trajectories were analysed by using Image-Pro Analyzer 7.0 software (Media Cybernetics Inc., Silver Spring, MD), and are 2D representations of a 3D transport. Movement of an individual particle centroid was transformed into time-dependent mean-square displacement (MSD), <Δr^2^(Δt)> = < Δx^2^ + Δy^2^>, where Δx and Δy are particle displacements in x and y directions, respectively, and Δt is the time scale over which the displacement was calculated^32,33^. By averaging MSDs from trajectories of many particles with identical Δt, ensemble mean-square displacement <MSD> was calculated for families of particles. Effective diffusivities (diffusion coefficients, D_eff_) were obtained from D_eff_ = MSD/4Δt, and ensemble effective diffusivities <D_eff_> calculated for families of particles by averaging D_eff_ values obtained for individual particles. The extent of particle interaction with the mucus network, and possible anomalous diffusion in mucus, was determined by fitting particle MSD vs. Δt to MSD = 4DΔt^α^, where D is the diffusion coefficient independent of time, and α is the anomalous exponent that has been used to classify the type of particle motion by providing insight into the magnitude of particle transport obstruction^32,34,35^. Individual particle motion type was characterised according to the α-value, where 0.8 < α < 1.0 is considered diffusive^36^, and α < 0.8 represents various degrees of immobilised transport. For each condition used in this study, a proportion of diffusive particles (% of total particles) was calculated. For the particles that were undergoing simple diffusion (i.e. the diffusive particles), apparent local microviscosity of mucus (η) was calculated using the Stokes–Einstein equation, D = k_B_T/6πηr, where k_B_ is the Boltzmann’s constant, T is an absolute temperature in Kelvin and r is the radius of diffusing particle^32,35^. Mean microviscosity was calculated by averaging viscosity values obtained for individual particles diffusing in a given type of mucus. All experiments were performed five times (i.e. for five individual mucus specimens) for each mucus source, and for mucus samples obtained from five individual pigs, piglets or humans. Each specimen was used for up to 25 min (after the 20 min incubation step). Results were shown as means ± SD and/or distributions of data from measurements, for each condition used. Statistical comparisons between two groups were made using a Student’s *t* test, and three (or more) groups were evaluated using 1-way ANOVA (significance level, *P* value < 0.05).

## Results and discussion

The first challenge for this study was to develop a method for collecting human small intestinal mucus during a colonoscopy procedure. This is because the procedure for bowel preparation before colonoscopy involves drinking substantial quantities of liquids. Moreover, completing the preparation on the day of colonoscopy is considered essential in clinical practise for good quality of the colonoscopy examination^24,25^. However, the liquid has to be eventually removed from the small intestinal lumen in order to allow mucus aspiration. Our observations in an audit of bowel preparation quality, from over 300 colonoscopies, has led to the conclusion that the optimal time after the completion of bowel preparation for obtaining mucus samples from the terminal ileum is 5–6 h. If sampling was attempted earlier, the physiological integrity of the aspirated mucus could be compromised by an excess of liquid in the ileum. The collection procedure has been described in detail in the Experimental section. Additionally, a video clip showing aspiration of the mucus has been included in the Supplementary Material (Video S1).

After the mucus from the human ileum had been collected, its structure and permeability were compared with those of the mucus secretions collected from the adult pig ileum and jejunum as well as from the jejunum of 2-week old piglets. We included these four different types of small intestinal mucus in the study in order to evaluate any influence of (1) the interspecies variation (i.e. adult human vs. adult pig; both for ileal mucus), (2) the intraspecies age variation (i.e. adult pig vs. piglet; both for jejunal mucus), and (3) the variation in the anatomical location of mucus (i.e. jejunal mucus vs. ileal mucus; both for adult pig) on its penetrability to particles.

Intestinal mucus is a porous gel^3,37,38^ and its permeability depends on the microstructural organisation of mucin glycoproteins and other components. Substantial differences in the microstructure of the fully-grown pig vs. piglet mucus were reported previously^9^, with the piglet mucus found more heterogeneous and fragmented than pig mucus. An explanation for this difference suggested it was caused by variations in the distribution of mucin-producing goblet cells and the glycation of secreted mucins during postnatal development of the mammalian gastrointestinal tract^39^. Similar differences in microstructure were observed consistently in this study between the jejunal pig and piglet mucus samples (Fig. 1B). Although the confocal microscopy allowed for evaluation of hydrated mucus samples, this could only be done at a microscale level. Therefore, we performed a SEM examination of mucus, which showed that fragmentation in polymer network of the piglet jejunal mucus might also exist at the sub-micron scale (Fig. 1C). The SEM also revealed a smaller pore size and tighter polymer network in pig jejunal mucus, which supports the previous conclusion of a more coherent microstructural organisation of the pig mucus^9^. Importantly for this study, we have not observed considerable differences in microstructure between the pig jejunal and ileal mucus and the human ileal mucus (Fig. 1B,C). However, the CLSM and SEM have only been used for a preliminary, qualitative evaluation of the mucus samples, and any further conclusions regarding the extent of microstructural differences or similarities, and whether they may influence mucus permeability, would be largely speculative. In our next step, we applied a multiple-particle tracking method in order to quantitatively compare all four mucus types in terms of microviscosity and particle transport characteristics.

Apart from the microstructural organisation of mucus, the ability of particulate matter to permeate the mucus barrier depends on the size and surface chemistry of particles^40,41^. Despite the common opinion that mucoadhesion of particles, such as drug nanocarriers, can improve the GI absorption of orally administered pharmaceuticals by increasing their retention in mucus^42,43^, more and more studies have shown that altering surface chemistry of particles in a way that allows them to penetrated mucus and avoid mucoadhesion can enhance drug delivery in the GI tract^19,44–46^. Mucoadhesive particles are vulnerable to clearance, as the mucus is ultimately cleared through muscular contractions such as peristalsis, resulting in particles moving distally with the intestinal contents^47^. Positively charged particles are especially prone to adhering to the GI mucus^46,48^, which is largely due to the electrostatic attraction to negatively charged mucus. The mucoadhesion can be reduced by enhancing the negative surface charge of particles, and one way for achieving this is by the adsorption of small intestinal bile salts (BS) to the particle surface. In several previous studies^21,28,49^, the adsorption of those anionic, physiological biosurfactants to the surface of partially digested emulsion droplets or carboxylate-modified latex beads has been shown to considerably improve their mucus penetrability. This included an increase in the fraction of particles able to diffuse in the mucus and an increase in the particle mean diffusion rate. The effect was assumed to be a result of increased electrostatic repulsion between the intestinal mucus matrix and the particles covered by negatively charged BS. In this study, we incubated 500 nm latex beads with 11 mM BS in order to reflect the average, postprandial, small intestinal concentration of BS in adult humans^31^. The treatment enhanced the negative charge of the beads, from − 18.8 ± 1.5 to − 49.5 ± 1.6 mV, and is consistent with what was observed recently^22^. Small intestinal mucus transport of nanoparticles has also been studied in the presence of other surfactants. Lock et al.^50^ showed that the passive diffusion of 200 nm beads through native porcine mucus was not significantly influenced by exposure to non-ionic Tween 80. The authors postulated that Tween micelles might diffuse through the mucus gel, minimally impacting its structure, and interact with hydrophobic regions of the mucus. More recently, Zhang et al.^51^ showed that the exposure of the porcine small intestinal mucus to Tween 80 reduced the bulk viscosity of the mucus and facilitated nanoparticle penetration through the mucus. The particulate diffusivity was enhanced to a larger extent after the mucus had been treated with sodium dodecyl sulfate, however the authors did not report on how the exposure to this anionic surfactant influenced the surface charge of diffusing nanoparticles.

**Figure 2:**
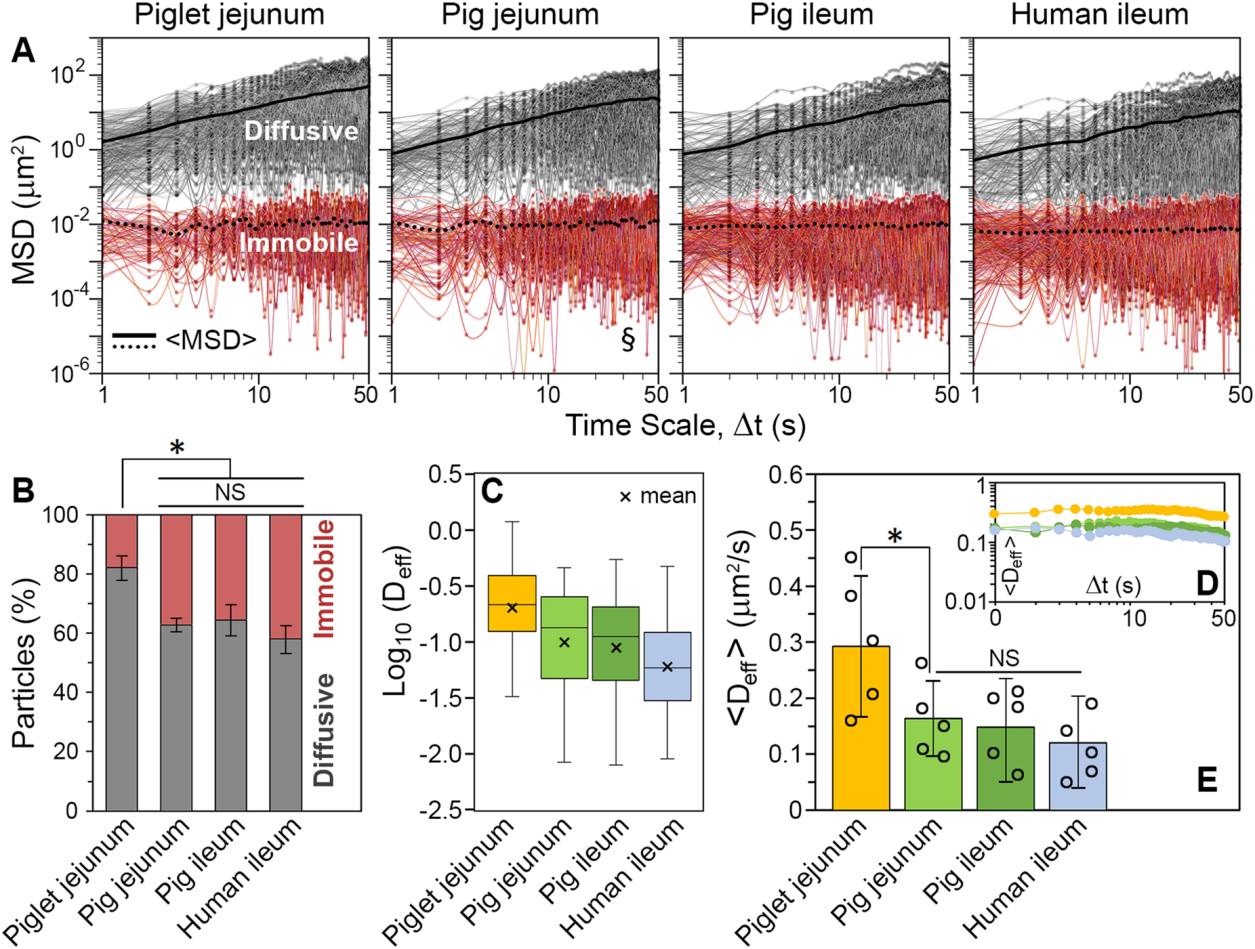
Ex vivo transport rates and distributions of 500 nm latex beads in the human and porcine small intestinal mucus. (**A**) Tracking of the motion of individual particles in mucus secretions collected from the piglet jejunum, the pig jejunum, the pig ileum and the human ileum showed variations in distance travelled by individual beads (individual mean-square displacement (MSD) values) over the time scale (Δt) of experiment. Individual particle trajectories were subdivided into fractions of diffusive (0.8 < α < 1.0) and immobile (α < 0.8) beads, and ensemble mean-square displacements (<MSD>) calculated for both fractions. (**B**) Proportions of the diffusive and immobile fractions of beads in the four types of mucus. (**C**) Distributions of the logarithms of mean diffusion coefficients (effective diffusivities, D_eff_) calculated only for individual diffusive beads (each, single mean D_eff_ value was calculated for each diffusive particle by averaging all individual D_eff_ values that were recorded for a particle over the entire Δt of 50 s). (**D**) Evolution of the ensemble diffusion coefficient (<D_eff_>) for diffusive fractions of beads as a function of Δt. (**E**) Comparison of mean <D_eff_> values (± SD). A single data point (○) represents the mean of all beads per a given subject. *N* = 5 for each type of mucus, with 110–150 beads per experiment (the exact numbers of beads analysed have been given in the “Supplementary Material”). * *P* < 0.05, by Student’s *t* test; NS, not significant (*P* > 0.05), by ANOVA). ^§^Measurements conducted under conditions similar to those previously reported^22^. All measurements were conducted at 37 ± 0.1 °C.

The BS-treated beads were used in multiple-particle tracking experiments to probe mucus permeability by assessing particulate diffusion and thus local microstructure. This method allows for dynamic measurements of the movement of individual particles and sub-populations within heterogeneous matrices^52^. The spacial positions of beads in mucus were recorded as a function of time and converted to mean-square displacement (MSD) from the initial location. Tracking of the motion of individual beads revealed a high level of heterogeneity within all mucus samples. In each case, a large population of particles that were able to diffuse in mucus was observed (Fig. 2A). However, the distances travelled by individual beads in mucus over the time scale of 50 s differed considerably. This might indicate variations in the viscosity between local microenvironments experienced by individual beads. The ensemble MSD (<MSD>) values calculated for these populations showed a steady increase in particle penetration over time (Fig. 2A). In all types of mucus, a population of beads that were immobilised by the mucus matrix (immobile particles) was also observed (Fig. 2A). The <MSD> values obtained for those populations showed no change in time, indicating the particles could not diffuse locally. Calculations of the anomalous exponent (α) for individual beads revealed that the population of immobile particles was least prolific in the piglet jejunal mucus (< 20% of all particles). This fraction was roughly two times smaller than in the other three types of mucus (Fig. 2B).

The evolution of MSD in time for each diffusive particle was converted into the effective diffusivity (diffusion coefficient, D_eff_(*t*)). A mean D_eff_ value was calculated for each diffusive particle by averaging all individual D_eff_ values that were recorded for a particle over the entire Δt of 50 s. Mean D_eff_ values were calculated individually, and only for diffusive particles (i.e. for the particles where α: 0.8–1.0; Fig. 2B). The mean D_eff_ values showed broad distributions for all four types of mucus analysed (Fig. 2C). This is an expected consequence of the heterogeneous structures of mucus (Fig. 1B,C), which must have impacted considerably on the distance a particle could traverse locally in mucus over the time scale of experiment (Fig. 2A). However, there were clear differences observed. In the piglet jejunal mucus, over 75% of individual diffusing particles returned D_eff_ values that were higher than the mean D_eff_ value recorded in any of the other three types of mucus (Fig. 2C). This indicates a looser arrangement of polymer network in the piglet mucus and confirms the observed difference in microstructural organisation of this mucus relative to the adult pig and human mucus secretions (Fig. 1B,C).

From the D_eff_(*t*) progressions of individual diffusing particles, the evolution of an ensemble diffusion coefficient (ensemble effective diffusivity, <D_eff_>) over time was also obtained by averaging D_eff_(*t*) values within fractions of diffusing particles in all the types of mucus (Fig. 2D). In each case, the <D_eff_> value was relatively constant over time, which indicated the particles were freely diffusing^53^. The comparison of mean <D_eff_> values (Fig. 2E) showed that the rates of particle diffusion in the jejunal mucus secretions differed by a factor of two between the piglet and the adult pig. The difference was significant (*P* < 0.05, by Student’s *t* test). A similar difference was observed in our previous study that investigated the impact of animal age and mucus composition (DNA contents, etc.) on the permeability of porcine jejunal mucus^9^. The motion of 500 nm latex beads with a weak, negative surface charge (ζ-potential ca. − 20 mV) was found to be almost completely hindered in the mucus obtained from fully-grown pigs, whereas in the mucus collected from piglets, a modest fraction of ca. 30% particles was able to diffuse. The two types of mucus were further treated in that study with DNase that hydrolysed extracellular DNA in the mucus, and considerably increased the fractions of freely diffusing particles to 64% and 77%, respectively. Importantly, the mean diffusion in the DNase-treated piglet mucus was twice as fast as in the DNase-treated pig mucus, indicating that other factors such as the structural organisation and concentration of mucin biopolymers might have played a crucial role in mucus penetrability. It is well established that diffusivity of nanoparticles can be substantially affected by a chemical composition (e.g. mucin and DNA contents) and microstructural organisation of mucus^4,22^. When comparing the adult pig vs. piglet mucus^9^, it was suggested the variations in mucus permeability and structure were caused by differences in the rates of mucin secretion and epithelial cell turnover during postnatal development. Similar discrepancies in particle diffusivity have been observed in the present study. Here, however, the motion of particles was not facilitated by selective hydrolysis of structural components of mucus, but by pre-incubating the particles with BS, which resulted in enhancing their negative surface charge and might have altered electrostatic interactions with the mucus matrix as discussed above. The additional ANOVA analysis and Tukey post hoc testing of all four mucus types included in this study discriminated between the piglet mucus vs. the three types of adult mucus (i.e. the pig jejunal and ileal mucus, and the human ileal mucus). The rates of particle diffusion did not differ significantly (*P* > 0.05) between these three types of adult mucus (Fig. 2E).

We have also compared the concentrations of mucus samples. Measurements of the dry weight content returned 19.1 ± 0.6% (w/w) for pig jejunal mucus, 19.4 ± 0.8% (w/w) for pig ileal mucus, and 19.9 ± 0.8% (w/w) for human ileal mucus. Statistical analysis of the results showed that the three groups did not differ significantly (*P* > 0.05). This suggests there might have been similar quantities of mucin/DNA in the adult pig and human samples available for mucus network formation. The dry weigh content of the piglet jejunal mucus was 16.2 ± 1.0% (w/w), which represents a 15–18% reduction relative to the three adult mucus samples. This might be one reason behind the different (i.e. more heterogeneous and fragmented) structural organisation of the piglet mucus (Fig. 1B,C), which allowed for the enhanced diffusivity (Fig. 2B–E). Significantly lower amounts of total mucin and DNA have recently been found in the small intestinal mucus of 5-day old rat pups relative to 21-day old rats^36^. The difference, caused by gut immaturity, was suggested to contribute to reduced barrier properties of the neonatal mucus. The ileal mucus of the 5-day old rat pups was shown in that study to be substantially more permeable to passively diffusing particles (200 nm PEG-, carboxyl- and amine-modified polystyrene beads) compared to the ileal mucus of 21-day old rats.

As for the primary goal of this study, comparing the permeability of mucus obtained from adult humans and pigs was more important. Mucus collected from fully-grown pigs is often used as a model system for a difficult-to-obtain human mucus in nutritional and pharmacokinetic studies looking at the mucus interactions of ingested foods and orally administered drug delivery systems in the gut^18–20,54^. Here, we have investigated colloidal transport of particles in the pig jejunal and ileal mucus and the human ileal mucus in order to assess whether the microstructural organisations of mucus from these three sources differ and how they impact on transport characteristics. Kirch et al.^55^ suggested that diffusibility of particles in mucus is dependent on mucus pore size. The pore diameter up to 200 nm was reported for purified porcine jejunal mucin network^56^. In the study on murine small intestinal mucus^3^, a similar average pore size of 200–220 nm was found for mucus gels in the jejunal and ileal segments. Despite this, there have been a number of reports showing that particles as large as 0.5–2 µm can diffuse freely in small intestinal mucus^3,9,22,28^. Those studies implied the mucus permeability is not only determined by the pore size, but that it also depends on interactions between the mucus and the diffusing particles as well as on the structural organisation of the mucus at various length scales, including nano- and microscale. Round et al.^56^ proposed a model of transport which involves weak interactions between lamellae of mucin network produced in mucus from individual mucin granules. According to the model, small non-mucoadhesive nanoparticles (i.e. smaller than the network pores) can pass freely through the lamellae, not interacting with the mucin until sterically trapped. Consequently, particles of larger diameters than the pore size can diffuse along transient channels between lamellae rather than through the networks. A similar, hierarchical mechanism of assembly of MUC2 mucin was reported more recently^4^. Mucin oligomers were found to form viscoelastic microscale domains via hydrogen bonding and Ca^2+^-mediated links, and the domains further aggregated to form a yield stress gel-like fluid. The authors proposed a microstructural model of mucin systems that accommodated the co-existence of both types of rheological behaviour.

The absorption of nutrients and drugs can take place along the whole length of the small intestine and its rate may depend on a local microstructure and other properties of the mucus layer. The thickness of jejunal and ileal mucus was compared before. In the rat, the distal ileal mucus layer was reported to be about four times thicker than the mucus in the proximal jejunum^10^. In another study on rats, Szentkuti et al.^57^ showed a very comparable mean thickness of the mucus layer in the two intestinal segments (ca. 90–100 µm). In the murine small intestine, the mean mucus thickness was found to decrease distally in Carnoy’s fixed samples, from ca. 40 µm in the duodenal section, and plateauing gradually at approximately 20 µm in the mid jejunum and ileum^3^. A similar lack of substantial difference in the thickness of jejunal and ileal mucus in the mouse has been reported by Ermund et al.^58^, although the mucus layers measured in that study were over 200 µm thick in both locations. It has also been observed that the layer thickness can fluctuate considerably between the fasting and the fed states of an animal^57^. This suggests that mucus microstructure may have greater importance in defining transport rates of nutrients, drugs or particles towards the intestinal epithelium than mucus thickness.

Up until now, the permeability of mucus from jejunum and ileum has not been compared. In this study, we did not observe a substantial difference in particle transport between the mucus samples collected from these two anatomical regions of the small intestine of adult pigs. The proportions of beads that were able to diffuse in the jejunal and ileal mucus secretions were 62.8 ± 2.3% and 64.3 ± 5.2%, respectively (Fig. 2B). These figures were only slightly higher than the 58.1 ± 4.7% found for the human ileal mucus (Fig. 2B). More importantly, tracking of diffusing particles proved that <D_eff_> values did not differ significantly (*P* > 0.05) between these three groups (Fig. 2E). This is in contrast to the piglet mucus vs. adult pig mucus comparison described above. This also suggests that the intramucus transport of particles in the adult human small intestinal environment can be simulated accurately by using the mucus collected from fully-grown pigs.

**Figure 3:**
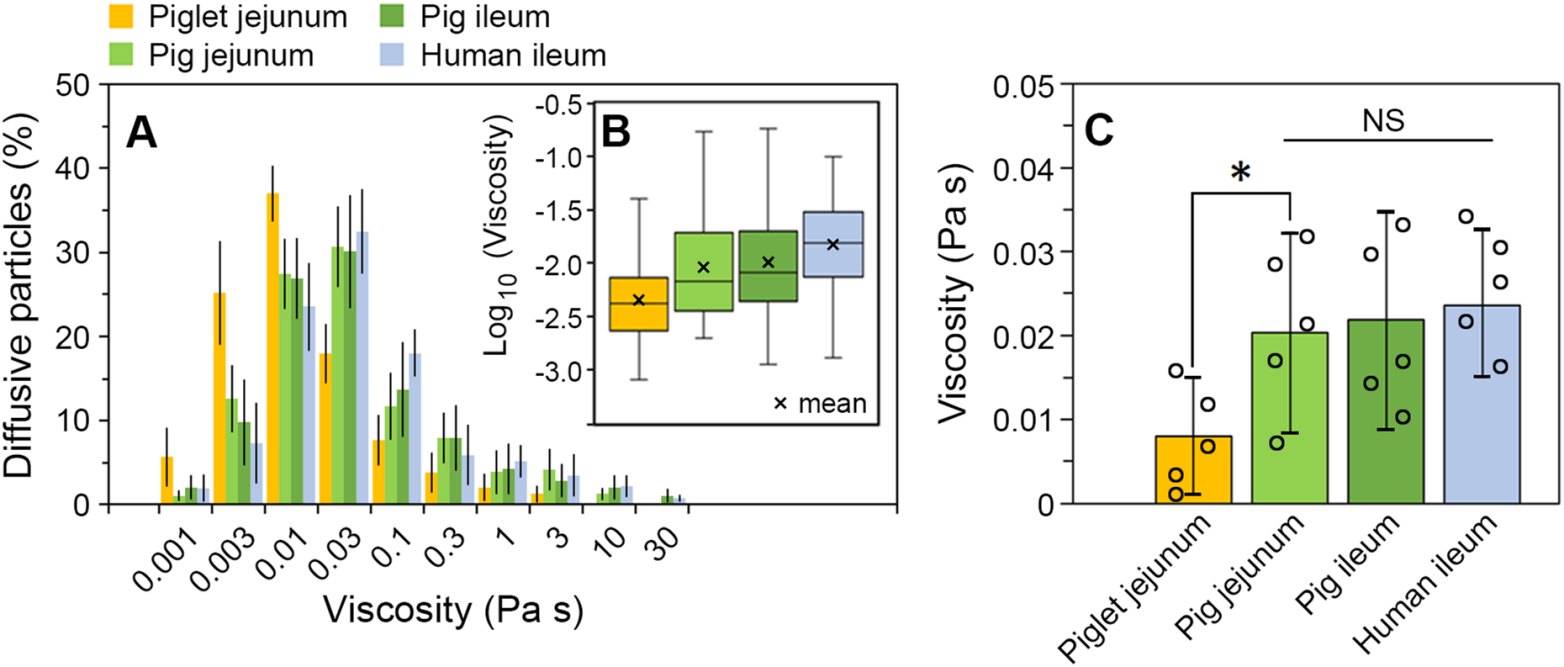
The Stokes–Einstein microviscosity of the human and porcine small intestinal mucus at 37 ± 0.1 °C. (**A**) Distribution of the apparent viscosity values of the piglet jejunal mucus, the pig jejunal mucus, the pig ileal mucus and the human ileal mucus as determined from diffusion of individual 500 nm latex beads (diffusive fractions; see Fig. 2B). (**B**) Box plot of the logarithms of individual viscosity values showing quartiles within each data set. (**C**) Comparison of the mean viscosity values (± SD) for all types of mucus analysed. A single data point (○) represents the mean of all beads per a given subject. *N* = 5, with 84–120 diffusive beads per experiment (*P < 0.05, by Student’s *t* test; *NS* not significant (*P* > 0.05), by ANOVA).

Tracking of individual particle motions has an advantage over estimation of ensemble averaged properties because it allows to reveal the heterogeneity within samples as well as how it differs between samples from different sources^54^. From the mean D_eff_ value obtained for each bead diffusing in mucus, the viscosity experienced locally by an individual bead at the microscale was calculated using the Stokes–Einstein equation (Fig. 3). The distributions of those microviscosity values are depicted in Fig. 3A. The viscosity ranged from 1 mPas to 10–30 Pas, and the broad extent of distribution was observed for every type of mucus. Taking into account that 18–42% (depending on the mucus type) of the total number of beads tracked in mucus were immobilised locally by mucus matrix (Fig. 2B), even higher viscosity could have been experienced by particles in those locations. The presence of BS was shown before to greatly enhance the diffusivity of particles in mucus by strengthening their negative surface charge and thus reducing mucoadhesion^21,28^. Therefore, mucoadhesion caused by insufficient electrostatic repulsion between some beads and the mucus network might be excluded from being a major cause of the immobilisation observed in the present study. It is more likely that those particles became entrapped in regions densely populated with aggregates of concentrated mucin (Fig. 1B) and their motion seized in confined spaces. Meldrum et al.^4^ studied diffusivities of 500 nm carboxyl-functionalised polystyrene microspheres in MUC2 mucin solution at various mucin concentrations. An abrupt transition in particle motion, from diffusive to sub-diffusive, was found by the researches after increasing mucin concentrations over 10 mg/mL, which suggested the viscoelastic behaviour of mucin over that threshold concentration restricted motion of the tracer particles. A sudden and substantial increase in microviscosity of mucin solutions has been shown in that study for mucin concentrations, c ≥ 15 mg/mL^4^. Taking all the above into account, the over two-fold increase in the numbers of immobilised particles in the adult pig and human mucus vs. the piglet mucus (Fig. 2B) seems to be the result of the fragmented microstructure of the piglet mucus, which appeared less coherent and easier to penetrate than the other types of mucus (Fig. 1B).

Those beads that were able to diffuse revealed substantial differences in the microviscosity between particular types of mucus. In the piglet mucus, 50% of diffusing beads experienced the viscosity ranging from 1 to 4.5 mPas (Fig. 3B). In the other types of mucus, roughly 25% or less of diffusing beads were tracked in regions with similarly low viscosity (Fig. 3B). Finally, the indication for a different microstructural organisation of the piglet jejunal mucus comes from its mean microviscosity value, which was more than two times lower than the value recorded for the adult pig jejunal mucus (Fig. 3C). The difference between these two groups was significant (*P* < 0.05, by Student’s *t* test). The additional ANOVA analysis and Tukey post hoc testing of all four mucus types discriminated between the piglet mucus vs. the three types of adult mucus (i.e. the pig jejunal and ileal mucus, and the human ileal mucus). In contrast to the significantly less viscous piglet mucus, the mean viscosities of pig jejunal and ileal mucus samples were very similar: 20.3 ± 11.9 mPas and 21.8 ± 13.0 mPas, respectively (Fig. 3C). The two values were comparable to the microviscosity of ca. 18.5 mPas recorded recently^22^ for freshly excised pig jejunal mucus, i.e. the mucus that was not stored frozen before examination. This supports the previous finding that a freezing and thawing of collected mucus does not considerably impact its microstructure with regard to particle penetrability^22^. Most importantly, the present work has shown that the Stokes–Einstein viscosity of the mucus collected from the jejunum or the ileum of adult pigs was similar to the viscosity recorded for the ileal mucus of adult humans. With the mean value of 23.4 ± 8.2 mPas, the viscosity of human mucus was not significantly different from the other two values obtained for the pig mucus secretions (*P* > 0.05; Fig. 3C).

## Conclusions

This study has investigated, for the first time, the permeability of human small intestinal mucus to sub-micron sized particles. In our previous work^22^, we found that the structure and microviscosity of porcine intestinal mucus is not considerably affected by detaching the mucus from the mucosal tissue and its storage that involved freezing and thawing. The present study is another crucial step required for developing human-relevant models for studying mucus transport. It provides evidence for a similar microstructural organisation of the adult pig mucus and the adult human mucus in terms of penetrability to particles. This is particularly important given the difficulty in obtaining sufficient quantities of human mucus samples. Our work validates the use of the pig small intestinal mucus in studies aiming to reliably mimic the transport of colloidal dispersions (e.g. food digesta, nanoparticulate drug carriers, etc.) through the mucus barrier under physiologically relevant conditions of the human small intestine.

## Supplementary information

Supplementary Information.

Supplementary Video S1.

Supplementary Video S2.

## Data Availability

All relevant data are within the paper.
