# Supplementary material for: Comparing the permeability of human and porcine small intestinal mucus for particle transport studies
Source: Sci Rep. 2020 Nov 20;10:20290. doi: 10.1038/s41598-020-77129-4 (PMC7679454; doi:10.1038/s41598-020-77129-4)
Supplement: Supplementary file 1 — Supplementary Information. [file 41598_2020_77129_MOESM1_ESM.pdf]

Comparing the permeability of human and porcine small intestinal mucus for particle transport studies

Lukasz Krupa[1], Balazs Bajka[2], Robert Staroń[1], Didier Dupont[3], Harjinder Singh[4], Krzysztof Gutkowski[1], Adam Macierzanka[3,4,5] *

[1] Department of Gastroenterology and Hepatology with Internal Disease Unit, Teaching Hospital No 1, Chopina 2, 35-055 Rzeszów, Poland
[2] Department of Nutritional Sciences, King's College London, London SE1 9NH, United Kingdom
[3] STLO, INRAE, Agrocampus Ouest, 35000 Rennes, France
[4] Riddet Institute, Massey University, Private Bag 11 222, Palmerston North 4442, New Zealand
[5] Department of Colloid and Lipid Science, Faculty of Chemistry, Gdańsk University of Technology, Narutowicza 11/12, 80-233 Gdańsk, Poland

*Correspondence and requests for materials should be addressed to A. Macierzanka
(email: adam.macierzanka@pg.edu.pl).

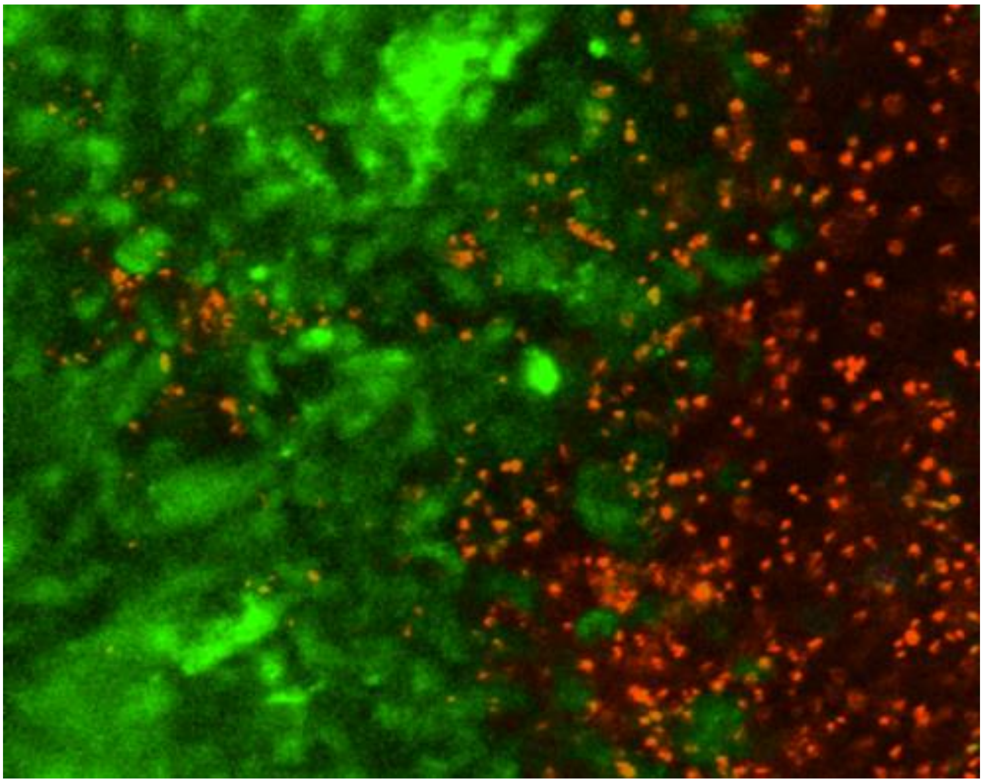

Fig. S1. A CLSM image (375×300 µm) showing the red-fluorescent, 500 nm latex beads that entered the pig jejunal mucus matrix (left-hand side of the image; mucus stained for mucin with WGA-Oregon Green) from the aqueous dispersion of beads (right-hand side of the image) during the incubation at 37 ± 0.1 °C (see also Video S2). The image was created using Image-Pro Analyzer 7.0 software (Media Cybernetics, Inc.; https://www.mediacy.com/imagepro).

Video S1. A representative video clip showing aspiration of the mucus from the human distal ileum during a colonoscopy procedure. The mucus collection method has been described in detail in the Experimental section of the manuscript.

Video S2. A representative video clip of the confocal time-lapse microscopy. The video (67×50 μm frame) shows transport of red-fluorescent, 500 nm latex beads inside the pig jejunal mucus (i.e. after they have entered the mucus matrix) captured over the course of 150s at 37 ± 0.1 °C and displayed at 15× speed.

<u>Supplementary information for</u> **Figure 2.**
The exact numbers of beads analysed for each specimen type (*N* = 5 for each type of mucus, with 110–150 beads per experiment):

- piglet jejunal mucus: 112, 129, 127, 147, 135,
- pig jejunal mucus: 132, 150, 113, 126, 144,
- pig ileal mucus: 140, 131, 151, 119, 130,
- human ileal mucus: 148, 146, 110, 138, 124.
